# Transgenic Alfalfa Plants Expressing the Sweetpotato *Orange* Gene Exhibit Enhanced Abiotic Stress Tolerance

**DOI:** 10.1371/journal.pone.0126050

**Published:** 2015-05-06

**Authors:** Zhi Wang, Qingbo Ke, Myoung Duck Kim, Sun Ha Kim, Chang Yoon Ji, Jae Cheol Jeong, Haeng-Soon Lee, Woo Sung Park, Mi-Jeong Ahn, Hongbing Li, Bingcheng Xu, Xiping Deng, Sang-Hoon Lee, Yong Pyo Lim, Sang-Soo Kwak

**Affiliations:** 1 Plant Systems Engineering Research Center, Korea Research Institute of Bioscience and Biotechnology (KRIBB), Daejeon, 305–4432, Korea; 2 Department of Horticulture, Chungnam National University, Daejeon, Korea; 3 State Key Laboratory of Soil Erosion and Dryland Farming on the Loess Plateau, Institute of Soil and Water Conservation, Chinese Academy of Science and Ministry of Water Resources, Northwest A & F University, Yangling, Shaanxi, P.R. China; 4 Grassland and Forages Division, National Institute of Animal Science, Rural Development Administration, Cheonan, Korea; 5 College of Pharmacy and Research Institute of Pharmaceutical Sciences, Gyeongsang National University, Jinju, Korea; 6 Department of Green Chemistry and Environmental Biotechnology, Korea University of Science & Technology, Daejeon, Korea; University of Tsukuba, JAPAN

## Abstract

Alfalfa (*Medicago sativa* L.), a perennial forage crop with high nutritional content, is widely distributed in various environments worldwide. We recently demonstrated that the sweetpotato *Orange* gene (*IbOr*) is involved in increasing carotenoid accumulation and enhancing resistance to multiple abiotic stresses. In this study, in an effort to improve the nutritional quality and environmental stress tolerance of alfalfa, we transferred the *IbOr* gene into alfalfa (cv. Xinjiang Daye) under the control of an oxidative stress-inducible peroxidase (*SWPA2*) promoter through *Agrobacterium tumefaciens*-mediated transformation. Among the 11 transgenic alfalfa lines (referred to as SOR plants), three lines (SOR2, SOR3, and SOR8) selected based on their *IbOr* transcript levels were examined for their tolerance to methyl viologen (MV)-induced oxidative stress in a leaf disc assay. The SOR plants exhibited less damage in response to MV-mediated oxidative stress and salt stress than non-transgenic plants. The SOR plants also exhibited enhanced tolerance to drought stress, along with higher total carotenoid levels. The results suggest that SOR alfalfa plants would be useful as forage crops with improved nutritional value and increased tolerance to multiple abiotic stresses, which would enhance the development of sustainable agriculture on marginal lands.

## Introduction

Soil salinization and desertification are two major environmental problems that greatly limit agricultural production worldwide [1]. Areas affected by salt and drought stress account for almost half of the world’s agricultural lands [2]. For most crops, high salinity and water deficit severely decrease crop growth, yield, and quality due to increased osmotic and oxidative stress. Due to the dramatic increase in the human population, together with increasingly serious environmental problems, it will be difficult for world agriculture to meet the world’s food and energy requirements in the future [3]. Therefore, it is necessary to develop novel crop cultivars with excellent environmental stress tolerance for use in sustainable agriculture.

Alfalfa (*Medicago sativa* L.), one of the most important legume forage crops, is widely cultivated throughout the world due to its significant economic value and excellent agricultural traits [4]. As one of the highest-yielding forage crops, alfalfa has outstanding nutritional quality, with high levels of protein, minerals, and vitamins as well as well-balanced amino acids. Moreover, the nitrogen-fixing ability of alfalfa reduces the amount of energy required for its production and improves soil structure. In addition, alfalfa has deeper roots than most crops. This deep, vigorous root system allows alfalfa to adapt to various environmental conditions and increases its capacity to prevent soil erosion. Thus, alfalfa is considered to be a potential forage crop for use in areas subjected to environmental stress such as drought and high salinity [5]. However, the pernicious effects of abiotic stress (such as salt and drought stress) still represent major limits to alfalfa production. To improve the adaptability of alfalfa to these environmental stresses, many studies focused on modifying various aspects of alfalfa plants, such as the activation of cascades of molecular networks involved in stress responses [6].

Carotenoids, a class of colorful pigments and important nutrients, are biosynthesized in the plastids (such as chloroplasts and chromoplasts) of plants [7, 8]. These multifunctional metabolites, which have powerful antioxidant activity, play critical roles in light collection, protection of photosynthetic systems, and abscisic acid (ABA) synthesis [9, 10, 11]. The accumulation of carotenoids in tobacco and *Arabidopsis* plants has resulted in enhanced tolerance to abiotic stresses including UV irradiation, high light, and salt stress [9, 12, 13].

The *Orange* gene (*Or*), which is involved in carotenoid accumulation, shares a high level of homology in many crops such as cauliflower, rice, tomato, and *Arabidopsis thaliana* [14, 15]. Introduction of the cauliflower *Or* gene induced the formation of chromoplasts and increased the carotenoid contents in transgenic potato tubers [16]. Transgenic rice callus expressing *AtOr* accumulate higher levels of carotenoids than the control [15]. We previously determined that the *Or* gene (*IbOr*) from orange-fleshed sweetpotato is not only involved in carotenoid accumulation, but it also functions in response to multiple abiotic stresses [11]. The expression of *IbOr* rapidly increases after NaCl, PEG, and H_2_O_2_ treatment. Transgenic sweetpotato calli overexpressing *IbOr* exhibit enhanced tolerance to salt stress, with increased carotenoid contents and antioxidant activity. Thus, the *Or* gene may be useful for developing valuable crops with enhanced tolerance to multiple environmental stresses and increased nutrient contents.

When designing an efficient expression system, it is important to select the proper promoter [17, 18]. We previously isolated and characterized the strong, oxidative stress-inducible sweetpotato peroxidase anionic 2 (*SWPA2*) promoter [19]. The *SWPA2* promoter induces higher levels of exogenous gene expression than the 35S promoter from the Cauliflower mosaic virus (CaMV 35S promoter) in response to various stress treatments, and it was successfully applied to several transgenic plants such as poplar, potato, and rice [20–22]. The *SWPA2* promoter can be worked at most organs including leaf and root in response to oxidative stress. Therefore, the stress-inducible *SWPA2* promoter is highly suitable for generating transgenic plants with enhanced tolerance to environmental stresses.

In this study, to improve the nutritional quality and environmental stress tolerance of alfalfa, we generated transgenic plants expressing *IbOr* under the control of the *SWPA2* promoter (referred to as SOR plants) through *Agrobacterium*-mediated transformation. The SOR plants exhibited enhanced tolerance to multiple abiotic stresses, along with increased accumulation of carotenoids. The results indicate that SOR alfalfa plants might be useful as forage crops for sustainable cultivation on marginal lands.

## Materials and Methods

### Construction of plant expression vector

*IbOr-Ins* cDNA was utilized to construct the expression vector used in this study due to its strong ability to increase carotenoid accumulation and salt tolerance in plants [11]. To construct the plant expression vector, a chimeric gene cassette, containing *IbOr-Ins* and the nos terminator (*NOSt*) under the control of the oxidative stress-inducible *SWPA2* promoter [19] with *Eco*RI and *Hin*dIII sites, was constructed and ligated into the corresponding sites of the pCAMBIA2300 binary vector, which harbors the *nptⅡ* gene as a selectable marker. The resultant vector, pS*IbOr-Ins*, was mobilized into *Agrobacterium tumefaciens* strain EHA105 for alfalfa transformation via a freeze–thaw method [23].

### Plant materials and transformation

*Medicago sativa* L. seeds (cv. Xinjiang Daye) were provided by Prof. Bo Zhang of Xinjiang Agriculture University, China [24]. The seeds were surface-sterilized with 0.5% mercuric chloride solution for 30 min, thoroughly rinsed 7–8 times with distilled water, and germinated on Murashige and Skoog (MS) medium (pH 5.7) [25] with half-strength vitamins and salt under a 16/8 h light/dark cycle, with a light intensity of 150 μmol m^-2^ s^-1^ and a relative humidity of 65% at 25°C. Five days after germination, the hypocotyls of seedlings were utilized for plant transformation with *A*. *tumefaciens* containing the *SWPA2* promoter-*IbOr* cassette.

The transformed calli were induced from infected hypocotyls and selected on Schenk and Hildebrandt (SH) medium [26] containing 2.0 mg l^-1^ 2, 4-dichlorophenoxyacetic acid (2, 4-D), 0.2 mg l^-1^ kinetin, 250 mg l^-1^ cefotaxime, and 50 mg l^-1^ kanamycin. Shoots were regenerated from the calli following transfer to MS medium containing 1.0 mg l^-1^ benzylaminopurine (BAP), 0.3 mg l^-1^ 1-naphthylacetic acid (NAA), 250 mg l^-1^ cefotaxime, and 50 mg l^-1^ kanamycin. Throughout the experiments, the cultures were maintained in a culture room at 25 ± 2°C under a 16 h photoperiod. Regenerated shoots were transferred to full MS medium for rooting. The rooted plantlets were transferred to soil after 1 week of acclimation in pots in the growth chamber.

### PCR analysis

Genomic DNA was extracted from the leaves of alfalfa plants as previously described [27]. PCR was conducted with purified genomic DNA in a PCR premix (Cat. no. K-2012, Bioneer, Korea) using a specific primer set designed based on the sequence of the *IbOr* gene and part of the *SWPA2* promoter to confirm the integration of *IbOr* (Table 1). The amplification reactions consisted of 94°C for 5 min (1 cycle), followed by 30 cycles (94°C for 45 s, 60°C for 45 s and 72°C for 1 min) and a final extension cycle of 7 min at 72°C. The PCR products were separated on a 1% agarose gel, stained with ethidium bromide, and visualized under UV light. All subsequent experiments were conducted on cloned plants from the T_0_-generation of transgenic plants.

**Table 1:** Gene-specific primers used for genomic and RT-PCR analysis.

Gene name	Primer sequences (5’–3’)
*SWPA2*-3'_F	Forward: GAAACCTTAGAGGCAATTCATGCA
*IbOr_*R	Reverse: CGTGGGTCATGCTCGCTTGCCATAGCCATC
*IbOr-*RT	Forward: ATCTCCATGGAAGGCTCAAATC
	Reverse: CGACGGATGAAGAAAAGGAG
*MsPSY* (KJ955630)	Forward: GCACCTGAATCCAAGGCTTC
	Reverse: GCATCTTCTCCAACATCTCTGA
*MsCHY-β* (KJ955631)	Forward: ACGGTGTTTGGGATTGCCTACA
	Reverse: TTGGTGAGCAGCAGCAACTCTT
*MsLCY-β* (KJ955632)	Forward: GCATTGAAGAAGATGAGCAGTG
	Reverse: ACCACCGATTCCAACAACTCT
*MsNCED* (KJ955633)	Forward: AGCCACACTATGTAGCCGTTA
	Reverse: TGACCAATCCAATGCCGTTAG
*MsActin* (JQ028730.1)	Forward: TCCTAGGGCTGTGTTTCCAAGT
	Reverse: TGGGTGCTCTTCAGGAGCAA

### Gene expression analysis

To activate the *SWPA2* promoter and induce *IbOr* expression, similarly sized plants grown in soil for 1 month were treated with 5 μM MV, 250 mM NaCl solution, or withholding water for 1 week. Leaves (from the same position) of alfalfa plants were utilized for RNA extraction. Total RNA was extracted using a GeneAll Ribospin Plant kit (GeneAll, Seoul, Korea) and treated extensively with RNase-free DNase I to remove any contaminating genomic DNA. For semi-quantitative and quantitative expression analysis of related genes such as *IbOr* and the *Actin* gene in alfalfa plants, total RNA (2 μg) was used to generate first-strand cDNA using Moloney murine leukemia virus (MMLV) reverse transcriptase from an RT-PCR kit (TOPscript RT Dry MIX) in accordance with the manufacturer’s instructions. For semi-quantitative RT-PCR analysis, the PCR conditions were as follows: an initial denaturation step at 94°C for 5 min (1 cycle), followed by 27 cycles (94°C for 45 s, 60°C for 45 s, and 72°C for 30 s) and a final extension cycle of 7 min at 72°C. The PCR products were separated and visualized as described for genomic PCR analysis. Quantitative real-time PCR (q-RT-PCR) was performed in a fluorometric thermal cycler (DNA Engine Opticon 2, MJ Research, USA) using Ever-Green20 as a fluorescence dye according to the manufacturer’s instructions. The alfalfa *Actin* gene was used as an internal control for analysis of gene expression. Transcript levels were calculated relative to the controls. Data represent the means and standard errors of three biological replicates. The expression levels of the *IbOr*, *Actin*, and alfalfa carotenogenic genes (*MsPSY*, *MsCHY-β*, *MsLCY-β*, and *MsNCED*) were analyzed by quantitative real-time PCR using the gene-specific primers listed in Table 1. The PCR product of each alfalfa carotenogenic gene was confirmed by sequencing analysis and further analyzed through alignment with other known plant carotenogenic genes.

### Methyl viologen (MV) treatment and ion leakage analysis

The oxidative stress tolerance assay was performed as previously described [28]. Four leaves collected from the same position on four alfalfa plants were floated in a solution containing 0.4% (w/v) sorbitol and 5 μM MV, placed in the dark for 12 h to allow diffusion of MV into the leaves, and subjected to continuous light (150 μmol m^-2^ s^-1^) treatment at 25°C. The loss of cytoplasmic solutes following MV treatment, based on the electrical conductance of the solution, was measured with an ion conductivity meter (model 455C, Istek Co., Seoul, Korea) over a time period ranging from 0 to 36 h and compared with the total conductivity of the solution following tissue destruction. The extent of cellular damage was quantified by ion leakage, which is an indicator of membrane disruption. The measurements were conducted in triplicate with three independent plants of each line.

### Salt and drought stress treatment

One-month-old alfalfa plants grown in a growth chamber at 25°C (60% relative humidity, 16/8 h [light/dark] photoperiod with light supplied at an intensity of 150 μmol m^-2^ s^-1^) were utilized for the abiotic stress tolerance assay.

For salt stress treatment, alfalfa plants were irrigated with a 250 mM NaCl solution every 2 days for 1 week. For dehydration treatment, the plants were irrigated with similar quantities of water through trays placed underneath the pots for 1 week, followed by withholding water for 7 days. The plants were then watered and allowed to recover from the drought conditions.

### Analysis of relative water contents

The relative water contents (RWC) were measured as described previously [21, 29]. The following formula was utilized: RWC (%) = [(FW-DW) / (TW-DW)] × 100, in which FW = immediate weight of freshly collected leaves, TW = turgid weight of leaves after incubation in water for 6 h at 20°C in the light, and DW = dry weight of the same leaves after drying at 80°C for 48 h. RWC was measured using the fourth fully expanded leaf from the shoot apical meristem.

### Analysis of chlorophyll contents

Chlorophyll contents were measured with a portable chlorophyll meter (SPAD-502, Konica Minolta, Japan) from the fifth intact, fully expanded leaves (counting from the shoot apical meristem) of individual plants. Total chlorophyll contents after stress treatment were compared with those under normal conditions.

### Analysis of lipid peroxidation

Lipid peroxidation was estimated by measuring the malondialdehyde (MDA) contents using a modified thiobarbituric acid (TBA) method [30, 31]. Approximately 0.1 g of leaf tissue was ground in 10 ml of 10% trichloroacetic acid (TCA) using a mortar and pestle. The homogenate was centrifuged at 10,000 rpm for 20 min. The reaction mixture (containing 2 ml of extract and 2 ml of TBA) was heated at 100°C for 30 min, quickly cooled on ice, and centrifuged again at 10,000 rpm for 20 min. The absorbance at 450, 532, and 600 nm was determined using an ultraviolet spectrophotometer (Spectronic, Genesys2, USA). The measurements were conducted in triplicate with three independent plants of each line.

### Analysis of H_2_O_2_ contents

For the H_2_O_2_ assay, alfalfa leaves (collected from the same position) were incubated in a 1 mg ml^-1^ solution of 3,3-diaminobenzidine (DAB)-HCl (pH 3.8) for 6 h at 25°C under continuous light according to the methods described previously [10, 32, 33]. The chlorophyll was removed via incubation at 80°C for 2 h in 80% ethanol.

### Analysis of proline contents

The free proline contents of alfalfa plants were measured spectrophotometrically as previously described [34, 35]. Leaf tissues were homogenized in 1.5 ml of aqueous sulfosalicylic acid (3%), and the residue was removed by centrifugation at 12,000 g for 10 min. One milliliter of the supernatant with 1 ml of acid-ninhydrin and 1 ml of glacial acetic acid was boiled in a water bath at 100°C for 1 h. After cooling the reaction mixture, 2 ml of toluene was added, and the mixture was vortexed vigorously and incubated at room temperature for 30 min until it separated into two phases. The upper phase (containing proline) was measured with an ultraviolet spectrophotometer (Spectronic, Genesys2, USA) at 520 nm using toluene as the blank. The proline concentration was quantified based on a standard curve using D-proline.

### Analysis of carotenoid contents

Carotenoids were extracted from leaves of 1-month-old alfalfa plants and analyzed using an Agilent 1100 HPLC (high-performance liquid chromatography) system (Hewlett-Packard, Palo Alto, CA, USA) as previously described [11]. All extraction procedures were performed under subdued light to avoid pigment degradation and loss. Twenty microliters of standard or sample was injected directly onto a YMC C_18_ carotenoid column (3 μm, 4.6 × 250 mm) with solvent A (MeOH- tert-butylmethyl ether [MTBE]-H_2_O [81:15:4]) and solvent B (MeOH-MTBE-H_2_O [6:90:4]) using a step gradient elution of 100% solvent A for the first 15 min, followed by 100% solvent A to 100% solvent B over the next 35 min. A conditioning phase (50–60 min) served to return the column to its initial state. The flow rate was 0.7 ml/min, and the column temperature was 30°C. The eluent was detected at 450 nm using a UV-visible detector. The HPLC-DAD system was operated via chemstation software (Hewlett-Packard). Carotenoids were quantified using an external calibration method. Carotenoid standards were purchased from CaroteNature (Lupsingen, Switzerland). The measurements for each line were conducted in duplicate with four biological repeats.

### Statistical analysis

Data were statistically analyzed by one-way ANOVA. Subsequent multiple comparisons were examined based on the least significant difference (LSD) test. All statistical analyses were carried out using the Statistical Package for Social Sciences (SPSS 12); statistical significance was set at * *P < 0*.*05* and ** *P < 0*.*01*.

## Results

### Generation of transgenic *IbOr* alfalfa plants

**Fig 1:**
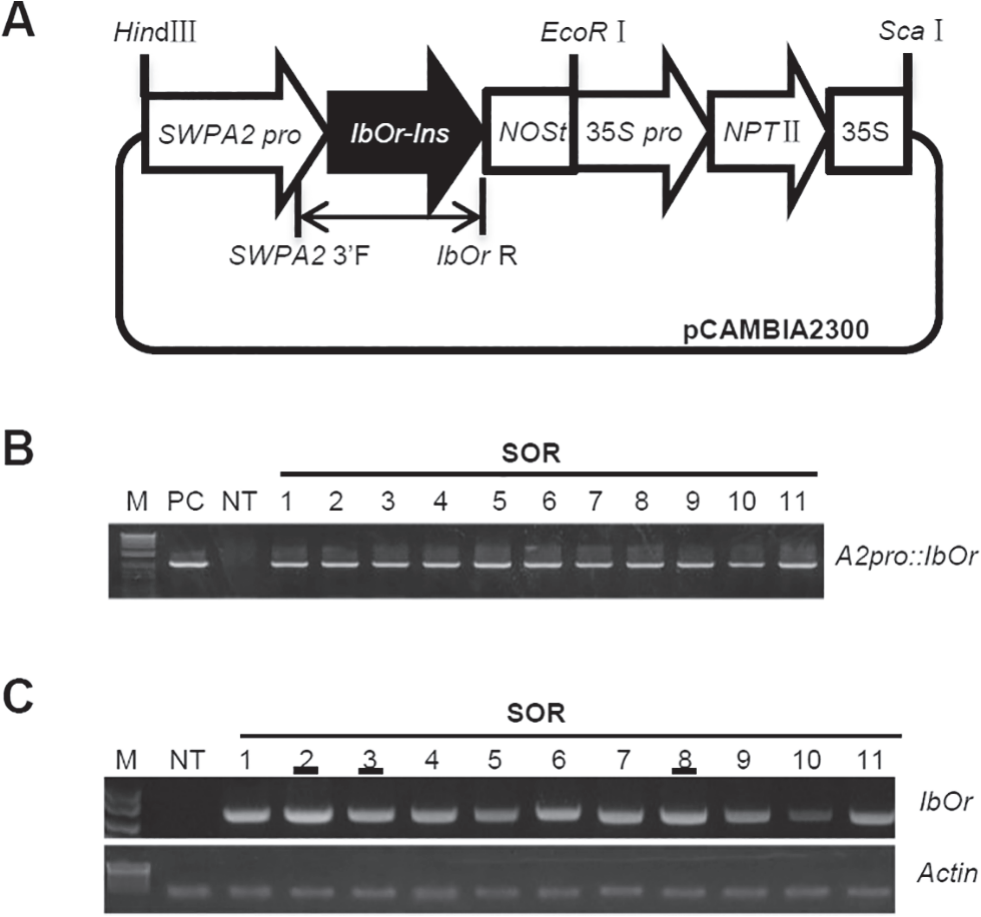
Development of transgenic alfalfa expressing *IbOr* under the control of the *SWPA2* promoter (SOR plants). (A) Diagram of the oxidative stress-inducible *SWPA2* promoter: *IbOr* construct used for alfalfa transformation. Vertical bar shows the primer set (*A2pro*::*IbOr*) used for genomic PCR analysis. (B) Genomic DNA PCR analysis using the *A2pro*::*IbOr* primer set. Numbers (1–11) represent independent transgenic lines. M, size markers; NT, non-transgenic plant; PC, positive control. (C) RT-PCR analysis of 11 lines expressing stable *IbOr* gene integration in transgenic plants following 2 h of 5 μM MV treatment.

Transgenic alfalfa plants expressing *IbOr* under the control of the oxidative stress-inducible *SWPA2* promoter (referred to as SOR plants) were successfully generated via *Agrobacterium*-mediated transformation (Fig 1A). Eleven kan-resistant alfalfa lines containing the *IbOr* gene were obtained and initially verified via genomic DNA PCR analysis using a specific primer set (Table 1). As shown in Fig 1B, the recombinant *IbOr* gene had been integrated into the genomes of all 11 independent transgenic alfalfa lines, while no integration was detected in non-transgenic (NT) plants. Regenerated alfalfa plants grown in a growth chamber for 1 month were then analyzed by semi-quantitative reverse transcription polymerase chain reaction (RT-PCR) using MV-treated leaf discs. The induced expression of *IbOr* was detected in all 11 transgenic lines, but not in NT plants. Among the 11 SOR lines, *IbOr* transcript levels were highest in lines SOR2, SOR3, and SOR8 (Fig 1C). These lines were, therefore, selected for further analysis.

### Enhanced tolerance to MV-mediated oxidative stress in SOR alfalfa

**Fig 2:**
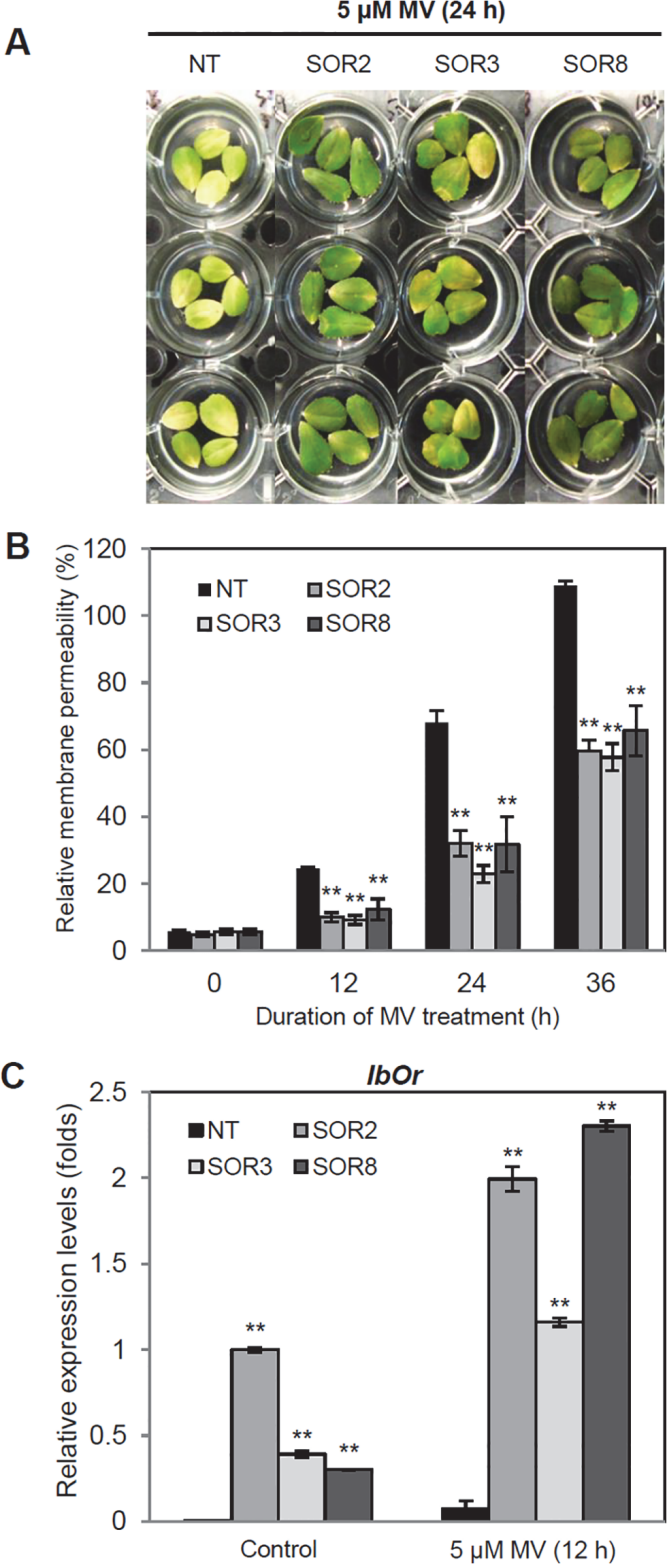
Effects of MV-mediated oxidative stress on leaves of SOR and NT plants. (A) Visible damage in leaves after 5 μM MV treatment for 24 h. (B) Ion leakage was measured after 0, 12, 24, and 36 h of MV treatment. Percentage of relative membrane permeability was calculated using 100% to represent the values obtained after autoclaving. (C) *IbOr* transcript levels after 12 h of 5 μM MV treatment. The expression levels of *IbOr* were normalized to that of the alfalfa *Actin* gene as the internal control. Data are expressed as the mean ± SD of three independent biological replicates. *Bars* labeled with *asterisks* show significant differences from that of NT at * *P < 0*.*05* or ** *P < 0*.*01* by *t*-test.

To investigate the MV-mediated-oxidative stress tolerance of the SOR plants, leaves (from the same position) of 1-month-old SOR and NT plants were treated with 5 μM MV solution for various periods of time (0, 12, 24, and 36 h). MV is a non-selective herbicide that induces the production of massive bursts of reactive oxygen species (ROS) in plants, which disrupts membrane integrity, leading to cell death. At 24 h following MV treatment, serious visible damage was detected in NT leaves, whereas SOR leaf discs exhibited only partial necrosis (Fig 2A). We quantified the degree of cellular damage based on solute ion leakage, a recognized indicator of membrane stability against oxidative stress [36]. The transgenic lines SOR2, SOR3, and SOR8 exhibited significantly lower levels of ion leakage (32%, 22.8%, and 31.7%, respectively) than NT plants (68.2%) at 24 h under MV treatment (Fig 2B). SOR plants displayed better membrane stability than NT plants under MV- mediated oxidative stress. We then performed q-RT-PCR to analyze *IbOr* expression in leaves treated with MV for 12 h. The *IbOr* transcript levels were significantly higher in transgenic lines SOR2, SOR3, and SOR8 than in NT plants under both normal and MV-treatment conditions (Fig 2C). The SOR plants exhibited higher *IbOr* expression after stress treatment than under normal conditions. These results indicate that the expression of *IbOr* increased the tolerance of SOR alfalfa plants to MV-mediated oxidative stress.

### Increased tolerance to salt stress in SOR alfalfa

**Fig 3:**
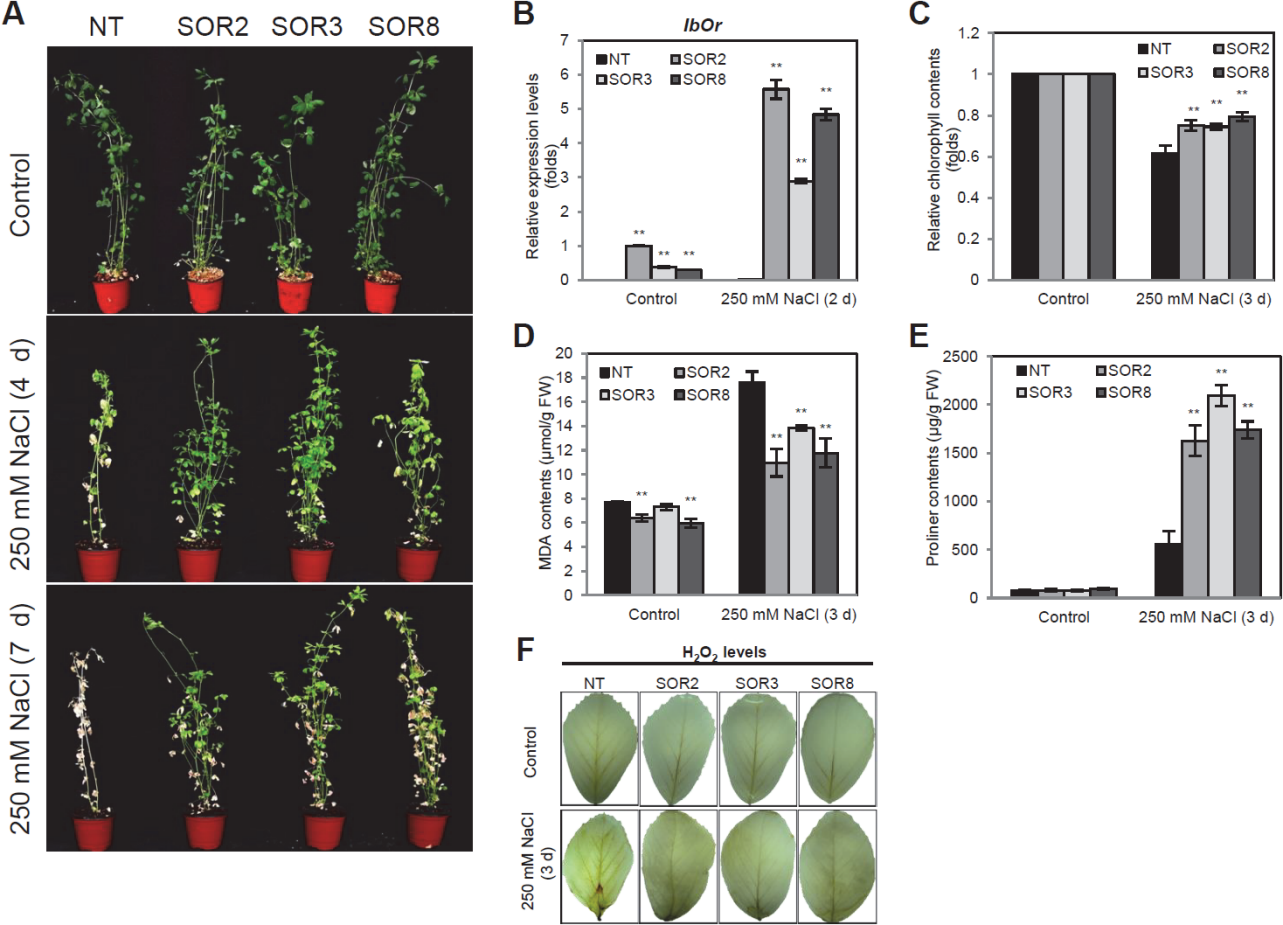
Enhanced tolerance to high salinity in SOR plants. (A) Plant growth under normal conditions (upper panel) and salt stress (250 mM NaCl) for 4 days (middle panel) and 7 days (lower panel). (B) Relative transcript levels of *IbOr* in leaves. (C) Relative chlorophyll contents of alfalfa plants after 3 days of salt treatment. (D) MDA contents in leaves after 3 days of salt treatment. (E) Proline contents of alfalfa plants after 3 days of salt treatment. (F) DAB staining for H_2_O_2_ accumulation in the third detached leaves after 3 days of 250 mM NaCl treatment. The values represent the mean ± SD of three independent replicates. Asterisks indicate a significant difference from that of NT at * *P < 0*.*05* or ** *P < 0*.*01* by *t*-test.

To analyze the salt stress tolerance of the SOR alfalfa plants, 4-week-old NT and SOR plants were irrigated with 250 mM NaCl solutions for 7 days. No obvious differences were observed between the NT and SOR lines under normal conditions. After 4 days of high salt treatment, the growth of NT plants was severely inhibited compared with the SOR lines. All three SOR lines grew well, with only a few slightly yellow leaves observed, whereas most leaves of NT plants showed severe wilting and chlorosis. At day 7 of NaCl treatment, the SOR lines exhibited less damage than the NT plants, which were almost dead (Fig 3A).

To confirm the levels of *IbOr* transcripts after salt stress treatment, leaves of NT and SOR plants subjected to NaCl treatment for 2 days were examined by q-RT-PCR. Under both non-stressed and stressed conditions, no expression of *IbOr* was detected in the NT plants, whereas the expression of *IbOr* in SOR plants was strongly induced under salt stress versus normal conditions (Fig 3B).

We measured the chlorophyll contents in the central regions of the fifth leaves (from the shoot apical meristem) of both NT and SOR plants in the absence or presence of salt treatment. There was no significant difference in chlorophyll contents between NT and SOR plants before salt stress treatment. After 3 days of high salt treatment, the chlorophyll contents of all SOR and NT plants were reduced; however, the three SOR lines maintained relatively high levels of chlorophyll (21–25% reduction), while a dramatic decrease in chlorophyll levels (34–46%) was detected in the NT plants (Fig 3C). MDA, a naturally occurring product of lipid peroxidation caused by accelerated ROS production [37], is an important indicator of the degree of cell membrane damage under stress conditions [38]. Under normal conditions, the MDA contents of SOR3 were similar to those of NT plants, while SOR2 and SOR3 had slightly lower levels of MDA. However, when treated with 250 mM NaCl for 3 days, higher levels of MDA were detected in NT plants than in the SOR lines (Fig 3D). The degree of cell membrane damage was greater in NT plants than in SOR plants under salt stress conditions. These results indicate that the presence of the *IbOr* gene can inhibit or eliminate the accumulation of ROS in the cell. Moreover, proline, an important compatible osmolyte [39], plays a critical role in plant adaptation to environmental stress. We therefore measured the proline contents of NT and SOR plants (Fig 3E). Under non-stress conditions, NT and all SOR lines had similar proline contents. However, after salt stress treatment, the SOR lines had 2.9–3.7 times higher levels of free proline than NT plants. In addition, the leaves of SOR alfalfa plants had lower levels of H_2_O_2_ than NT plants. SOR leaves of salt stress-treated plants exhibited little color change after DAB staining, while those of stressed NT plants were brown due to high H_2_O_2_ levels (Fig 3F). These results indicate that *IbOr* expression increases salt stress tolerance in alfalfa plants.

### SOR alfalfa exhibits increased drought stress tolerance

**Fig 4:**
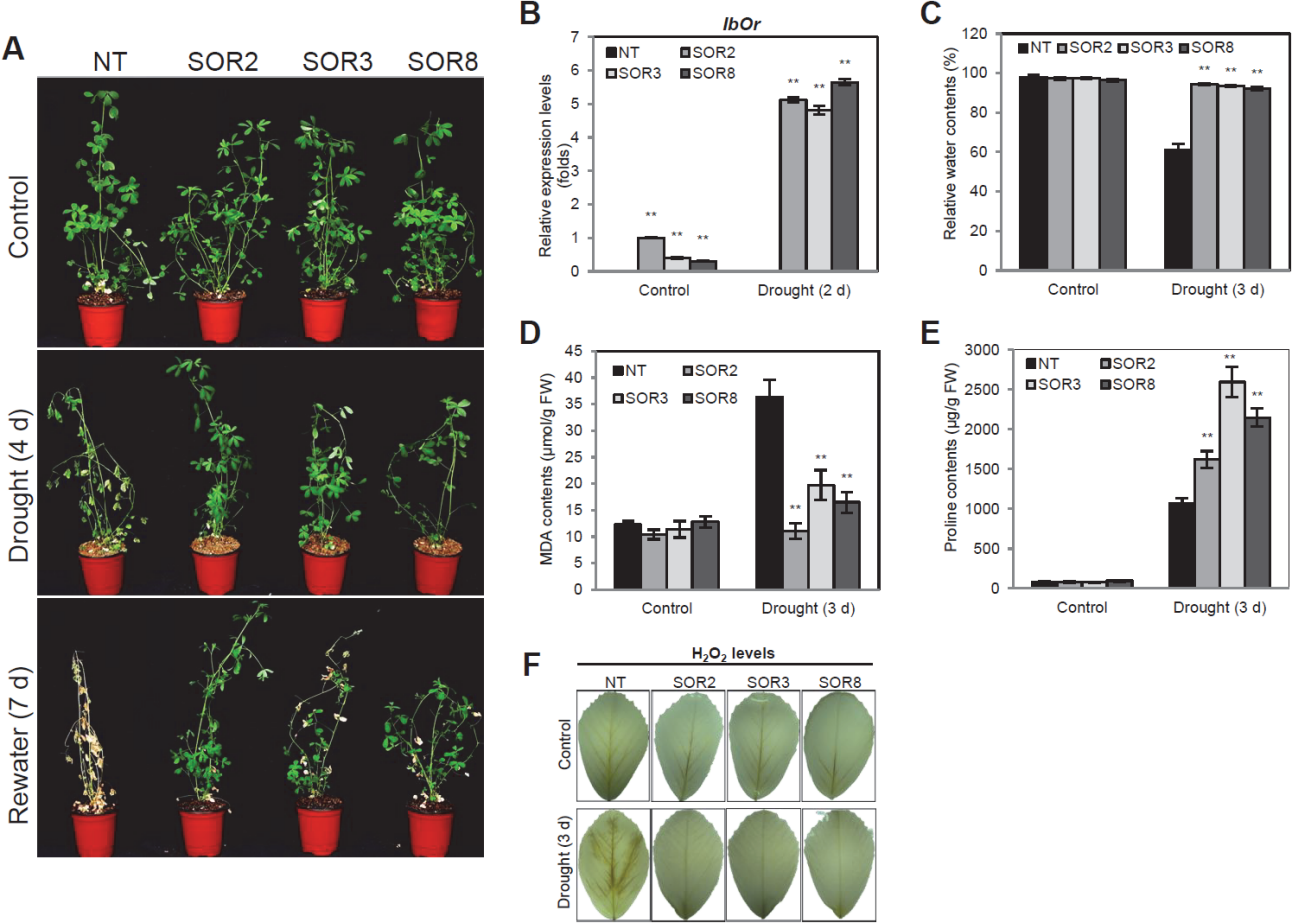
Enhanced tolerance to drought stress in SOR plants. (A) Phenotypes of 1-month-old NT and SOR plants before treatment (upper panel), after withholding water for 4 days (middle panel), and recovered phenotype after re-watering for 7 days (lower panel). (B) Transcript levels of *IbOr* after withholding water for 2 days. (C) RWC (%) in leaves after 3 days of water withholding. (D) MDA contents in leaves after 3 days of drought treatment. (E) Proline contents in leaves after 3 days of drought treatment. (F) DAB staining for H_2_O_2_ accumulation in the third detached leaves of alfalfa plants after 3 days of drought treatment. Values represent the mean ± SD of three independent replicates. Asterisks indicate a significant difference from that of NT at * *P < 0*.*05* or ** *P < 0*.*01* by *t*-test.

To evaluate the drought stress tolerance of SOR plants, 1-month-old NT and SOR plants were subjected to water deficit for 7 days. Before withholding the water supply, the plants were irrigated with similar quantities of water for 1 week; no obvious differences were observed between SOR and NT plants. After 4 days of withholding water, we observed severe wilting of the NT plants, while the SOR plants exhibited less wilting. When the plants were re-watered after drought stress treatment, all SOR lines recovered successfully, with only a few withered leaves, whereas NT plants were almost dead and failed to recover from dehydration conditions (Fig 4A). *IbOr* transcript levels were significantly higher in SOR plants than in NT plants after drought treatment (Fig 4B). To assess the degree of drought stress, the RWC values, a good indicator of the turgidity maintained by plants, were estimated using leaves from alfalfa plants before and after withholding water for 3 days. After 3 days of drought stress treatment, severe water loss was observed in the leaves of NT plants, with only 61.2% water content, while SOR2, SOR3, and SOR8 plants maintained significantly higher RWCs: 92.1%, 93.6%, and 94.3%, respectively (Fig 4C). In addition, SOR plants exhibited significantly lower MDA levels than NT plants after 3 days of drought stress treatment (Fig 4D). The degree of cell membrane damage under drought stress was greater in NT plants than in SOR plants. Under control conditions, the levels of free proline were similar between NT and SOR plants. However, higher levels of free proline were detected in the three SOR lines than in NT plants after 3 days of drought stress treatment (Fig 4E). Moreover, the SOR lines did not exhibited distinct color changes in stress-treated leaves following DAB staining, whereas a dark brown color through the leaf veins was observed in NT plants due to high levels of H_2_O_2_ under drought stress conditions (Fig 4F). These results indicate that the presence of *IbOr* increases drought stress tolerance in SOR plants.

### Analysis of carotenoid contents and expression of carotenoid biosynthetic genes under drought stress

**Fig 5:**
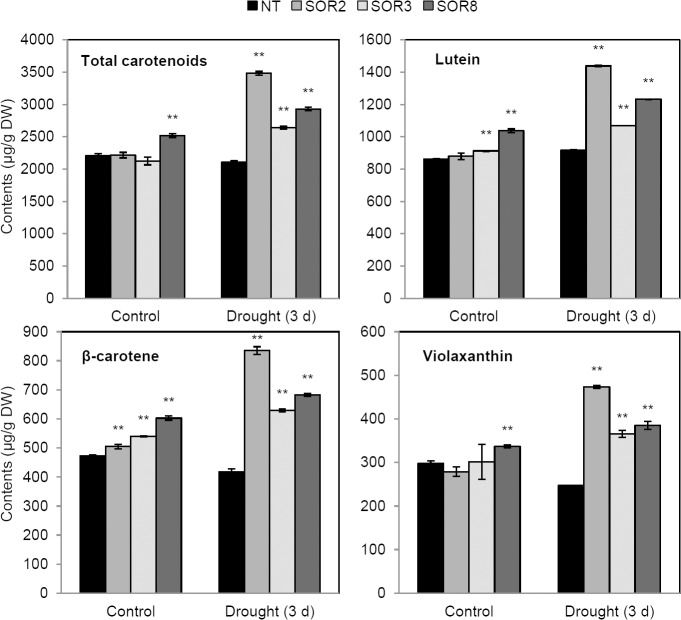
Quantitative HPLC analysis of total carotenoid contents and carotenoid compounds in NT and SOR plants. All levels are expressed as the mean (average content in grams dry weight) ± SD of two independent determinations with four biological repeats. Asterisks indicate a significant difference from that of NT at * *P < 0*.*05* or ** *P < 0*.*01* by *t*-test.

We previously reported that transgenic white-fleshed sweetpotato calli expressing *IbOr* had higher carotenoid levels than the control [11]. To investigate whether the expression of *IbOr* increases carotenoid accumulation in transgenic alfalfa plants, we performed quantitative analysis of carotenoids in leaves from 1-month-old NT and SOR plants using HPLC (Fig 5). Under non-stress conditions, the total carotenoid levels in SOR2 and SOR3 plants were similar to those of NT plants, while SOR8 had higher levels of total carotenoids including lutein, β-carotene, and violaxanthin. When subjected to drought stress for 3 days, the total carotenoid contents in all three SOR lines were approximately 1.3–1.7 times higher than those of NT plants. Among carotenoids, the levels of lutein and violaxanthin in SOR plants were 1.2–1.6 and 1.5–2.0 times those of NT plants, respectively, while the β-carotene levels in SOR plants were 1.51–2.0 times those of NT plants under drought stress conditions.

**Fig 6:**
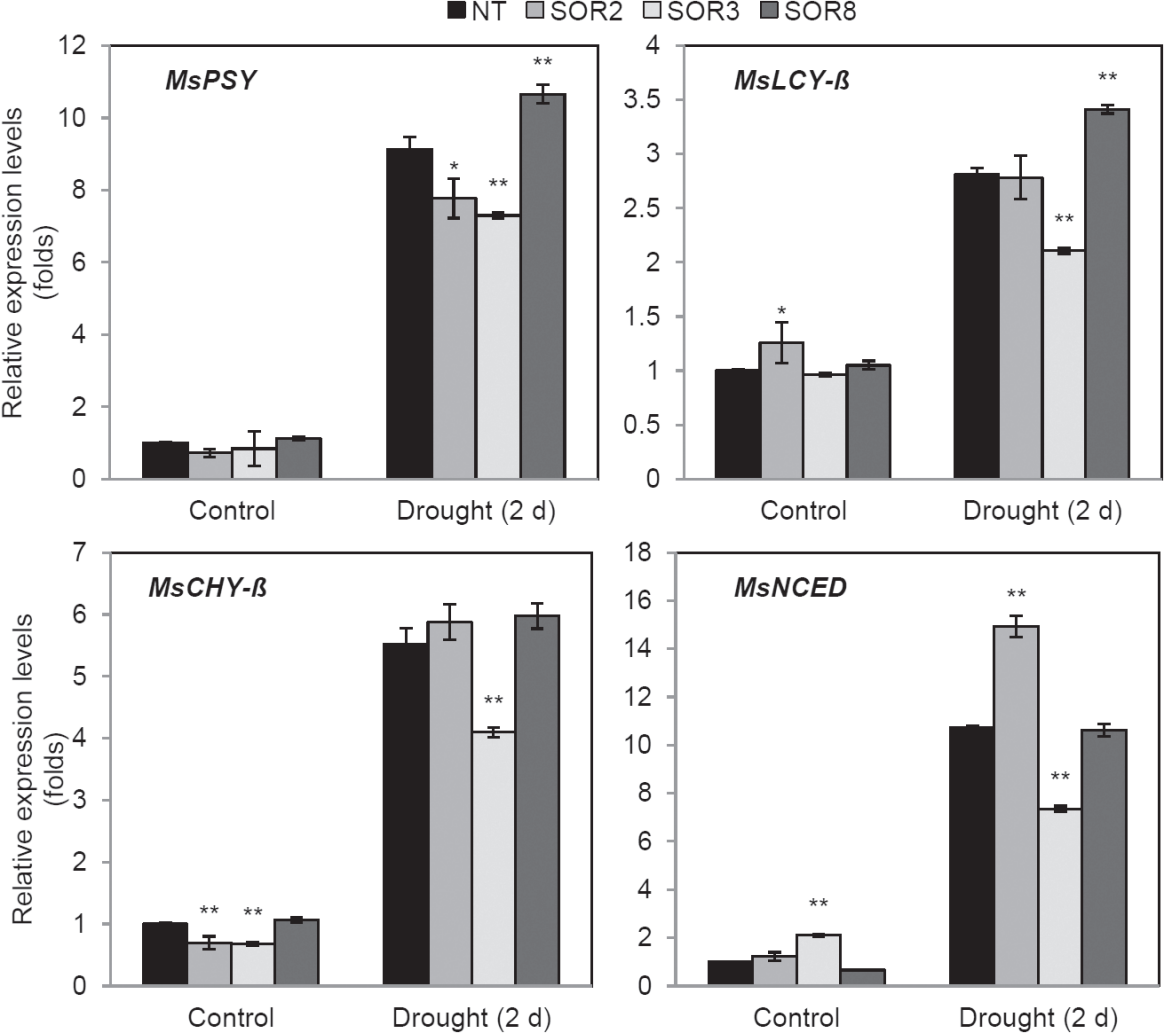
Transcript analysis of carotenoid biosynthetic pathway genes in NT and SOR plants under drought stress. Leaves (from same position) of plants treated with drought stress for 2 days were utilized. The expression level of each gene was normalized to that of the *Actin* gene of alfalfa as the internal control. *MsPSY*, phytoene synthase; *MsCHY-β*, *β-*carotene hydroxylase; *MsLCY-β*, lycopene *β*-cyclase; *MsNCED*, 9-cis-epoxycarotenoid dioxygenase. The values represent the mean ± SD of three independent biological replicates. Asterisks indicate a significant difference from that of NT at * *P < 0*.*05* or ** *P < 0*.*01* by *t*-test.

To investigate whether the increased accumulation of carotenoids in SOR alfalfa under drought stress was due to the presence of the *IbOr* transgene, we estimated the transcript levels of four genes (*MsPSY*, *MsLCY-β*, *MsCHY-β*, and *MsNCED*) associated with carotenoid metabolism in alfalfa leaves by q-RT-PCR. After 2 days of drought stress treatment, the expression of these four carotenogenic genes increased in both NT and SOR alfalfa plants compared with those under normal conditions (Fig 6). Interestingly, there was little relationship between increased carotenoid accumulation and the expression of carotenogenic genes in SOR plants.

## Discussion

There has been a dramatic increase in the area of saline-alkali and arid lands due to the expanding global population and inappropriate human activities such as overgrazing, deforestation, and improper soil and water management. Aridity and high soil salinity are the most serious environmental factors that reduce the yield and quality of crops on marginal lands worldwide [6]. The yield and quality of alfalfa, the most economically important legume forage crop, are also severely limited by drought and salt stress [5]. Therefore, novel alfalfa cultivars with stronger adaptability to these types of abiotic stresses are needed. The alfalfa cv. Xinjiang Daye used in this study was selected based on its good adaptability and yield performance [24]. We successfully developed transgenic alfalfa lines expressing *IbOr* under the control of the oxidative stress-inducible *SWPA2* promoter (Fig 1). The SOR plants exhibited enhanced tolerance to multiple abiotic stresses such as oxidative, salt, and drought stress. In addition, the carotenoid levels of SOR plants increased after drought stress treatment. These results demonstrate that *IbOr* can potentially be used to improve the agricultural traits of alfalfa to improve this crop’s adaptability to various environmental stresses.

Recent advances in plant biotechnology have led to the production of transgenic plants with excellent resistance to many abiotic stresses as well as improved biomass or nutritional value. We recently reported that the expression of *AtNDPK2* in transgenic alfalfa plants significantly increased their growth and improved their tolerance to oxidative, salt, and drought stress [40], indicating that the use of multifunctional transgenes is a highly efficient technique for crop improvement. The *IbOr* gene from orange-fleshed sweetpotato is strongly responsive to abiotic stresses and confers increased tolerance to salt stress [11]. In the current study, the expression of *IbOr* under the control of the stress-inducible *SWPA2* promoter in SOR alfalfa was also highly induced under stress conditions including oxidative, salt, and drought stress (Figs 2C, 3B and 4B). *IbOr* was expressed in SOR plants under normal conditions, perhaps due to the growth environment in pots and the sensitivity of the *SWPA2* promoter to abiotic stress. These results are compatible with our previous findings in transgenic sweetpotato calli expressing *IbOr* [11].

Abiotic environmental stresses cause excessive accumulation of ROS, which results in reduced growth, productivity, and quality in many crop plants [1]. *IbOr* transgenic sweetpotato calli exhibit higher antioxidant activity than NT calli [11]. In the current study, all three SOR alfalfa lines exhibited enhanced tolerance to oxidative stress, with less visible damage and loss of ion leakage than NT plants after 24 h of MV treatment (Fig 2A and 2B), indicating that *IbOr* increases the stability of cell membranes in SOR alfalfa. Moreover, SOR plants maintained higher levels of chlorophyll and salinity tolerance than NT plants throughout the salt stress period (Fig 3A and 3C). Kim et al. [11] found that transgenic *IbOr* sweetpotato calli exhibited lower levels of H_2_O_2_ production than control calli. We obtained similar results for SOR alfalfa plants under salt and drought stress treatment (Figs 3F and 4F), indicating that the ROS scavenging antioxidant ability was enhanced in these plants.

In plants under salt and drought stress, increased ROS production results in the accumulation of MDA due to membrane lipid peroxidation [41]. Stress-induced membrane damage and cell membrane stability are efficient criteria used to assess the degree of stress tolerance in plants [42]. Our results show that transgenic alfalfa plants expressing *IbOr* exhibited less cellular membrane injury, with markedly lower MDA contents, than NT plants (Figs 3D and 4D). Simultaneously, plants often accumulate compatible osmoprotectants, such as free proline, which protect them from stress [39, 40]. In the current study, SOR alfalfa plants under salt and drought stress exhibited a distinct increase in proline accumulation compared with NT plants (Figs 3E and 4E); this increase might contribute to the increased salt and drought tolerance of transgenic alfalfa. Increase of proline contents may be just because NT is dying and then produce less proline than transgenic lines. Despite their deep root system, the growth of most alfalfa cultivars is limited by drought stress [40]. We found that *IbOr* increased the viability of SOR plants under stress conditions, suggesting that this gene will be useful for improving plant tolerance to multiple abiotic stresses.

Carotenoids are important sources of nutrients, powerful antioxidants, and precursors of ABA biosynthesis [10, 33]. Since carotenoids play significant roles in the stress tolerance of many plants, metabolic manipulation of carotenoid biosynthesis was utilized to increase the abiotic stress tolerance and nutritional contents of crops such as sweetpotato and tobacco [10, 11, 13, 33]. Expression of the *Or* gene, which is highly conserved in many crops, results in carotenoid accumulation and chromoplast differentiation [11, 14, 15, 43, 44]. The cauliflower *Or* gene induces the formation of chromoplasts and large amounts of β-carotene in non-pigmented curd tissues [14]. Bai et al. [15] reported that overexpression of *AtOR* in rice callus increases the level of total carotenoids twofold. We previously demonstrated that *IbOr* accelerates the accumulation of carotenoids in transgenic sweetpotato calli and tubers through inducing high expression of carotenogenic genes [11, 44]. The current results show that the total carotenoid contents, as well as the levels of lutein, β-carotene, and violaxanthin, were higher in SOR alfalfa plants than in NT plants after drought stress treatment (Fig 5). These results, combined with the increased expression of *IbOr* in SOR plants under stress conditions (Fig 4B), suggest that the enhanced tolerance of SOR plants to abiotic stresses might be attributed to the high levels of carotenoids in these plants due to increased *IbOr* expression under stress conditions. In addition, the expression of the cauliflower *Or* gene in transgenic potato tubers induces the upregulation of proteins such as heat shock proteins and glutathione *S*-transferases. Heat shock proteins, which function as chaperones, participate in protein folding or unfolding and protein assembly or disassembly, and have unique physiological functions such as enhancing cellular membrane stability in plants [45], while glutathione *S*-transferases are involved in responses to stress, hormones, and developmental changes [46]. Therefore, the increased expression of these functional proteins might contribute to the enhanced stress tolerance of organisms expressing the cauliflower *Or* gene. In addition, transgenic potato expressing *IbOr* exhibits increased carotenoid accumulation and enhanced tolerance to oxidative and salt stress [47]. The increased levels of carotenoids in SOR plants contribute to less damage of chlorophyll compared to NT plants under salt stress, since carotenoids play important roles in photosynthesis.

However, *IbOr* expression did not markedly affect the transcript levels of carotenoid biosynthesis genes in transgenic alfalfa plants under drought stress conditions (Fig 6), which indicates that there was no relationship between stimulative carotenoid accumulation and the expression of carotenoid biosynthesis genes in transgenic *IbOr* alfalfa. Recent studies suggested that increased carotenoid accumulation in transgenic *Or* organisms is due to enhanced sink strength rather than the increased transcript levels of carotenoid biosynthesis genes [11, 14, 16]. Moreover, the increased carotenoid biosynthesis in transgenic *Or* organisms might be associated with alterations in the binding activity of this gene to its target protein [11, 14, 48]. In transgenic potato tubers, the expression of the cauliflower *Or* gene increases the stability of phytoene synthase, a key enzyme in the carotenoid biosynthetic pathway [48], during cold storage. However, more powerful evidence is needed to clarify the physiological mechanism of IbOr protein in more detail. Therefore, further studies are currently underway to increase our understanding of the precise function of *IbOr*.

In conclusion, three transgenic alfalfa plants expressing *IbOr* under the control of the stress-inducible *SWPA2* promoter were successfully generated and characterized. The SOR plants exhibited enhanced tolerance to multiple abiotic stresses and increased carotenoid accumulation. The results suggest that the expression of *IbOr* not only improves crop quality, but it also increases the tolerance to multiple environmental stresses in alfalfa plants. We anticipate that SOR alfalfa plants will be useful as forage crops for the development of sustainable agriculture on marginal lands, which may help resolve global environmental and food problems in the future. We are planning to investigate the agricultural characters of *IbOr* alfalfa plants on marginal lands, such as desertification areas and the Loess plateau in China.
